# Moving beyond cytotoxicity in cancer immunotherapy: embracing tumor microenvironment remodeling for durable control

**DOI:** 10.1038/s41416-025-03133-y

**Published:** 2025-08-04

**Authors:** Nicholas Koelsch, Masoud H. Manjili

**Affiliations:** 1 Department of Microbiology and Immunology, Virginia Commonwealth University (VCU) School of Medicine, Richmond, VA, USA; 2 VCU Massey Comprehensive Cancer Center, Richmond, VA, USA

**Keywords:** Immunoediting, Immunological surveillance

## Abstract

The quest for a curative cancer immunotherapy remains elusive, hindered by a longstanding focus on tumor cell elimination through cytotoxic mechanisms. However, mounting evidence points to an underappreciated dimension of immune function: its capacity for tissue remodeling and homeostasis, which can shape a tumor-inhibitory microenvironment. This perspective review highlights the adaptation model of immunity, which reframes the immune response as a dual force capable of both preserving and disrupting tissue integrity. Central to this model is Signal IV, a novel pathway in which self-reactive immune cells interact with adaptation receptors (AdRs) on tissue cells through adaptation ligands (AdLs) on immune cells. This interaction activates anti-apoptotic pathways in target cells, enabling immune responses to promote tissue survival and homeostasis even in the presence of cytotoxic mediators. Crucially, the downregulation of AdRs in stromal cells, while preserved in malignant cells, creates a tumor-promoting microenvironment, whereas the reverse fosters tumor rejection. This paradigm challenges conventional approaches by shifting the focus from tumor cell destruction to restoring tissue integrity, offering a revolutionary framework for immunotherapy. By targeting the AdR–AdL axis to reprogram the tumor microenvironment, the adaptation model proposes a transformative strategy for harnessing immune responses to achieve durable cancer control.

## Introduction

Over the past century, cancer immunotherapy has reshaped the field of oncology, delivering unprecedented survival benefits and, in some cases, durable remissions. Landmark breakthroughs – from Coley’s pioneering work with bacterial toxins to modern monoclonal antibodies, immune checkpoint inhibitors (ICI), and CAR-T cell therapies – have leveraged the immune system to selectively target and eliminate cancer cells. Yet, despite these advances, the goal of a curative immunotherapy capable of life-long, relapse-free survival remains elusive, with many patients experiencing recurrence following initial remission [1]. This persistent limitation reflects an incomplete theoretical foundation underpinning immunotherapy development. Traditional approaches primarily target cytotoxicity, aiming to replicate pathogen-focused immune responses characterized by IFN-γ, TNF-α, and granzyme/perforin production. However, unlike pathogens, cancer arises from the body’s own cells  and often triggers self-reactive, tissue homeostatic immune responses that maintain tissue integrity - but may also inadvertently support tumor growth.

Accumulating evidence suggests that effective tumor control may depend on immune mechanisms that actively support tissue homeostasis, creating a microenvironment less conducive to tumor growth. Addressing this challenge requires moving beyond simple tolerance or suppression of self-reactive T cells, as suggested by the integrity model of immunity [2]. Instead, the adaptation model of immunity proposes that self-reactive immune cells play a dynamic role in maintaining tissue stability and functional integrity [3]. For instance, macrophages participate in remodeling of extracellular matrix (ECM) [4], conventional T cells are involved in neurogenesis and neuronal function such that depletion of CD4^+^ T cells resulted in impaired learning [5]. Myelin basic protein (MBP)-specific T cells—detected in healthy individuals [6, 7] - enhance hippocampal neurogenesis and improve spatial learning [8, 9], suggesting a constructive role for self-reactive T cells in tissue function. Similarly, IFN-γ-producing meningeal T cells prevent aberrant CNS hyperexcitability, indicating a role for tissue-supportive, self-reactive immune responses in the brain [10]. Also, meningeal IL-4 producing Th2 cells contribute to learning and memory [11]. In the mammary gland, IFN-γ producing Th1 cells inhibited branching by affecting luminal differentiation while Th2 cells increase branching [12]. In the lymph node feed arteriole of mice, conventional CD4^+^ T cells could maintain vascular function at a steady state [13]. Such homeostatic function of effector T cells has been viewed as another specialized T cell population as Tx cells [14], yet neither the self-nonself (SNS) model nor conventional negative selection in the thymus adequately accounts for the existence of these self-reactive T cells. Although conventional inflammatory T cells typically exhibit cytotoxic functions, their paradoxical outcomes in tissue homeostasis can be regulated by signals that preserve tissue viability. In this process, adaptation receptors (AdRs) on target tissues bind with adaptation ligands (AdLs) on inflammatory T cells, promoting resilience during inflammatory homeostasis [3, 15].

The immune system’s tissue remodeling functions exemplify a critical yet overlooked principle. While tissue-resident T memory cells (T_RM_) are present across various tissues and are suggested to play a role in maintaining tissue homeostasis [16], prevailing theoretical models that narrowly focus on the cytotoxic functions of T cells have hindered the discovery of their homeostatic mechanisms. This oversight has obstructed a deeper understanding of T_RM_’s pivotal role in tissue remodeling and preserving integrity, revealing a fundamental gap in our approach to immune biology. Additionally, amphiregulin-producing Th9 cells mediate tissue repair in allogeneic hematopoietic stem cell transplantation, balancing graft-versus-leukemia effects while mitigating graft-versus-host disease [17]. These findings underscore that focusing solely on cytotoxicity risks undermining tissue homeostasis, potentially fueling secondary cancers. It was reported that breast cancer patients who received cytotoxic chemotherapy are five times as likely as the general population to develop treatment-related AML [18]. For cytotoxic immunotherapy, secondary cancers were reported following ICI or CAR T cells therapy [19, 20], or after autologous hematopoietic cell transplantation in children [21]. This paper argues for a transformative approach in cancer immunotherapy, one that prioritizes homeostatic immune functions over purely cytotoxic responses. By optimizing integrative, stabilizing immune mechanisms, we could redefine cancer treatment, advancing toward sustained tumor control and moving closer to a genuinely curative approach in oncology.

## Current limitations of cancer immunotherapies

Current cancer immunotherapies extend life but fall short of delivering a cure. Clinical data reveal that bispecific antibodies targeting CD3 and tumor-associated antigens, such as CD19 or CD20, can induce effective T cell-mediated killing in lymphomas. However, the therapeutic responses remain limited; for example, only 36.4% of patients with aggressive Non-Hodgkin lymphoma (NHL) achieve complete or partial responses, with a median response duration of 7.8 months [22]. There are at least 16 bispecific antibody therapies currently undergoing clinical trials [23]. CAR-T cell therapies targeting CD19 offer significant promise in hematologic cancers, yet progression-free survival after one year in relapsed/refractory patients remains around 47% [24]. For multiple myeloma, CAR-T cells targeting B cell maturation antigen (BCMA) show initial efficacy, but relapses are common, with a median progression-free survival of 11.8 months in one study [25]. In a phase II clinical trial targeting the same antigen, an 18-month progression-free survival rate of 66.8%, though cytokine release syndrome occurred in 97.9% of patients [26]. These therapies face challenges such as second primary malignancies [27], cardiovascular toxicities affecting nearly 20% of patients [28], and the development of resistance mechanisms, including antigen downregulation [29]. Despite these challenges, CAR-T therapies like Tisagenlecleucel - approved by the FDA for refractory leukemia and relapse cases - achieved a 3-year relapse-free survival rate of 48% in eligible patients [30]. A recent follow-up data in relapsed and/or refractory acute lymphoblastic leukemia (ALL) patients suggest development of a common disease resistance mechanism, including downregulation/loss of CD19 antigen in 30–70% of patients who have recurrent disease after treatment [31]. Similarly, downregulation or loss of BCMA expression in ALL patients being treated with BCMA targeted CAR-T cells [32]. Although targeting multiple antigens may overcome a single antigen loss, they cannot offer complete efficacy. For instance, a meta-analysis of 12 trials using CAR-T cells that target CD19 alongside anti-B cell maturation antigen found a median overall survival of 26.63 months [33]. In fact, tumor escape and immune evasion mechanisms are largely driven by the TME, significantly impacting T cell–based immunotherapies. The TME’s complexity demands strategies that can modify this environment holistically, reshaping it to support immune responses that reinforce tissue homeostasis rather than merely pursuing direct tumor destruction. By focusing on restructuring the TME, we might counteract immune escape more effectively, achieving sustained outcomes and enhancing the efficacy of current immunotherapies.

ICIs have become essential in treating advanced NSCLC and other metastatic cancers, particularly in patients with high PD-L1 expression. For instance, pembrolizumab extends median progression-free survival to 10.3 months in PD-L1 positive NSCLC patients compared to 6 months with chemotherapy [34]. An open-label, multicenter, randomized, controlled, phase 3 study conducted at 87 centers in 16 countries demonstrated median overall survival of 32·7 months with pembrolizumab versus 15·9 months with ipilimumab after a median follow-up of 57·7 months [35]. In patients with metastatic melanoma resistant to anti-PD-L1 monotherapy, combination of anti-CTLA4 plus anti-PD-1 resulted in a median 3·0 months progression-free survival (PFS) compared to anti-CTLA4 alone with 2·6 months PFS [36]. Nevertheless, approximately 50% of resected stage II-IV melanoma patients develop recurrent disease within 5 years despite adjuvant anti-PD-1 therapy [37]. The most effective results of a combined anti-PD-1 and anti-CTLA4 immunotherapy was reported to be a 7-year intracranial progression-free survival in 42% of patients with active asymptomatic melanoma brain metastasis which appeared to keep the tumor under control rather than eliminating it [38]. This report suggests that when tumors enter the state of progression-free dormancy, they might be controlled by immunotherapy, because dormant cells are less likely to escape immunotherapy. However, the authors did not look at the state of dormancy by Ki-67 staining.

## Questioning the SNS model in current cancer immunotherapies

For the last 70 years, the SNS model has dominated this field, guiding research, shaping data analysis, and informing therapeutic development. The SNS model posits that immune cells tolerate tumors because they arise from self-tissues, only becoming immunogenic when they express mutations or neoantigens that signal them as “semi-nonself.” This framework has shaped current therapies, such as neoantigen vaccines and allogeneic hematopoietic stem cell transplants that aim to trigger graft-versus-tumor responses. However, clinical outcomes suggest that this model may not be sufficient. Peripheral tolerance mechanisms within the TME often suppress these immune responses, allowing tumors to evade eradication and ultimately limiting therapeutic effectiveness.

This raises a crucial question: could our dependence on the SNS model be hindering true progress in cancer therapy? If current approaches grounded in this framework yield only incremental survival gains, a paradigm shift may be necessary to unlock curative outcomes. This new paradigm would need to reframe immune-tumor interactions, not merely as a binary battle between self and nonself, but as a complex interplay of immune and tissue dynamics demanding innovative strategies. To transcend the limitations imposed by the SNS model, future immunotherapies may need to prioritize the restoration of tissue homeostasis within the TME, aiming to create conditions unfavorable for tumor growth rather than relying solely on tumor destruction. Such an approach could leverage the body’s natural homeostatic immune responses to reshape the TME and enhance immune resilience against malignant cells. This shift in perspective - from viewing immune cells as “tumor destroyers” to “tissue stabilizers” - holds the potential to move beyond the current boundaries of incremental survival benefits, paving the way for more sustainable, curative strategies in cancer treatment.

## Self-reactive immune responses participate in tissue homeostasis without causing any harm

There is a growing body of literature stemming from pre-clinical and clinical data on the presence of self-reactive T cells that are involved in tissue homeostasis without causing autoimmunity [39]. For instance, in the intestinal tract, tissue-resident γδ T cells produce barrier protective cytokines and growth factors, thereby actively participating in maintaining the homeostasis and barrier integrity of the intestinal epithelium [40]. Also, inflammatory T cells producing IFN-γ and TNF-α co-operate to promote proliferation of IEC through AKT-β-catenin, with prolonged interactions with IEC inducing the Wnt inhibitor Dkk1, thereby leading to apoptosis [41]. Such sequential proliferation and apoptosis of IEC is required to maintain epithelial homeostasis. These inflammatory T cells exhibit microbiota reactivity, and produce IL-17A for promoting mucosal barrier function [42, 43]. In mice, IFN-γ produced by NKT cells has been reported to regulate intestinal epithelial cell homeostasis, with the ablation of NKT cells or CD1d in vivo leading to altered epithelial cell proliferation [44]. In humans, CD8^+^ T_RM_ cells produce amphiregulin (AREG) that interacts with the epidermal growth factor receptor (EGFR), to promote epithelial cell regeneration such that blocking EGFR signaling or the cytokines IFN-γ and TNF resulted in the inhibition of this tissue remodeling process [45]. In fact, human CD8^+^ T cells, tumor-infiltrating lymphocytes and CAR T cells have both cytotoxic and tissue regeneration potential [45].

In the brain, tissue-resident memory T cells expressing the CD69 activation marker as well as producing IFN-γ and TNF-α have been detected in the healthy humans [46]. In the CNS, IFN-γ producing meningeal T cells prevent aberrant hyper-excitability [10] while TNF-α producing cells regulate oligodendrocyte cell survival, myelin formation and repair [47]. Interestingly, while TNF-α antagonists are beneficial to patients with Crohn’s disease and rheumatoid arthritis, they exacerbate MS [48]. This could be because of the predominance of cerebral TNFR2, which is linked to anti-apoptotic Bcl-xL [3], making TNF-α as a CNS protective cytokine [3]. TNFR2 lacks a death domain and activates pro-survival pathways through the recruitment of TRAF2 and subsequent activation of NF-κB involving PI3K and PKB/Akt [49, 50]. It was demonstrated that neuronal TNFR1 participate in demyelination while neuronal TNFR2 signaling provides neuroprotection [51]. Interestingly, TNFR2 deficiency results in female-biased spontaneous autoimmune CNS demyelination in myelin oligodendrocyte glycoprotein-specific TcR transgenic mice [48].

Studies with anti-TNF therapies in psoriasis reveal that TNF-α inhibition can impair healing, indicating the cytokine’s role in skin repair and homeostasis [52]. Skin-resident T cells and dendritic epidermal γδ T cells (DETCs) engage in tissue homeostasis and repair following acute UV radiation by secreting IL-17 to induce genes mitigating DNA damage in keratinocytes [53]. Similarly, DETCs modulate immune responses in skin homeostasis and repair by reacting to and secreting insulin-like growth factor 1 [54].

In the mammary glands, IFN-γ producing Th1 cells inhibited branching by affecting luminal differentiation while Th2 cells increase branching [12]. Also, conventional CD4^+^ T cells maintain vascular function at a steady state [13]. Tissue-resident macrophages express Lyve-1, a receptor for the ECM component hyaluronan, thereby participating in ECM remodeling [4, 55]. They also regulate epithelial cell division, along with mammary organoid development and branching through production of TNF-α, and subsequent activation of PI3K and key molecules in cell division, namely Cdk1 and Cyclin B1 [56]. Even in regard to disseminated cancer cells from breast tissue that remain dormant in lungs for prolonged periods, alveolar macrophages are able to keep them in check through TGFB2 signaling, which can be reawakened after loss of its receptor [57]. Both macrophages and T cells are implicated in mammary tissues for modulating cellular turnover and immune surveillance under homeostatic conditions. Tissue-resident T cells express the early activation marker, CD69, in the absence of any infection or damage [58], suggesting their role in tissue homeostasis [59].

## Central and peripheral adaptations: all somatic cells including malignant cells are adapted to tolerate inflammatory immune responses by expressing adaptation receptors

**Fig. 1:**
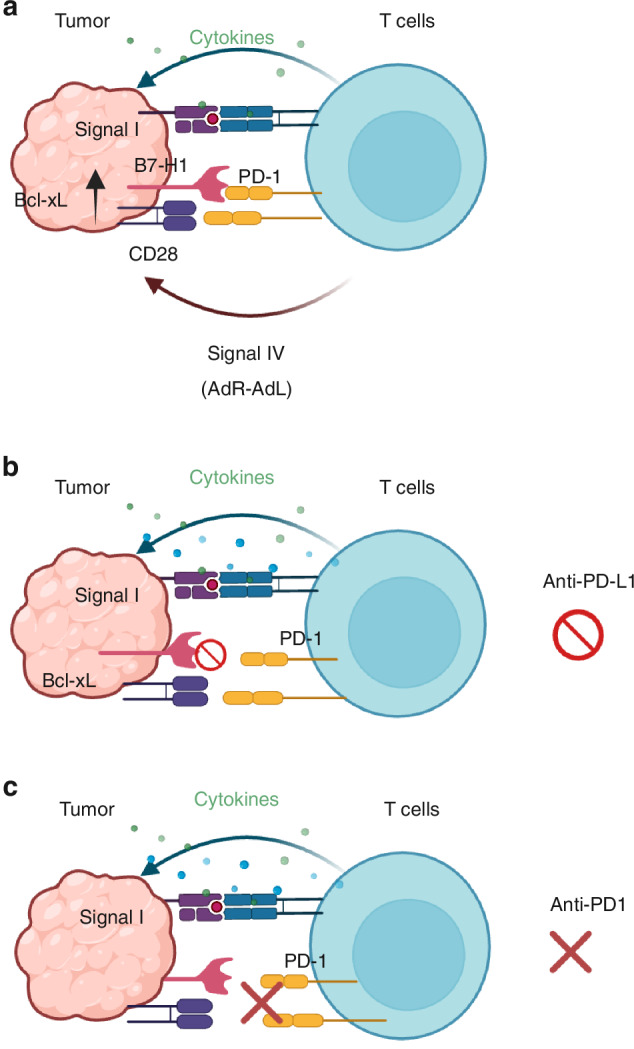
The role of signal IV in the outcome of anti-tumor T cell responses as well as the efficacy of ICI. **A** Anti-tumor T cells that recognize the peptide–MHC (pMHC) complex on tumor cells produce cytokines such as IFN-γ, which upregulate PD-L1 (B7-H1) expression on tumor cells. PD-L1 then engages PD-1 on T cells, initiating ‘Signal IV’, a feedback mechanism through which tumor cells upregulate anti-apoptotic Bcl-xL to resist T cell-mediated killing, while T cells simultaneously reduce IFN-γ production. **B** Blockade of signal IV by anti-PD-L1 antibody results in partial anti-tumor effect by inhibiting PD-L1’s engagement with PD-1, but tumor CD28 can still receive survival signal through engaging with PD-1 on T cells. **C** Blockade of signal IV by anti-PD-1 inhibits its engagement with both PD-L1 and CD28, resulting in a more effective tumor inhibition.

Self-reactivity of the immune response that participate in tissue homeostasis without causing autoimmunity, cannot be explained by the SNS model. The adaptation model proposes that self-reactive T cells are adapted through central and peripheral adaptations. During central adaptation, T cells pass through two tiers of positive selection (Table 1). First, cortical positive selection ensures MHC-restriction and elimination of T cells that fail to recognize self-peptide-MHC (pMHC) or signal I (pMHC-TcR interaction). Then, during medullary positive selection, defective T cells that cannot survive their activation upon receiving co-stimulatory signal II, are eliminated. On the other hand, functional T cells capable of receiving survival signals during co-stimulatory signal II are positively selected [3, 60], and are capable of mounting the anti-apoptotic Bcl-xL downstream of CD28 costimulation [61, 62]. In fact, cortical positive selection and medullary positive selection result in the adaptation of functional T cells that are capable of surviving signal I and signal II, respectively. These two tiers of positive selections ensure self-reactivity of T cells for participating in homeostasis of different tissues and maintaining the tissue integrity (Table 1). During peripheral adaptation, somatic cells in the lungs, liver, kidneys, heart, pancreas, thymus, anterior chamber of the eye, and placenta [63] that are exposed to self-reactive T cells exhibit AdR expression (Table 2). For instance, tissue-resident T effector/memory cells express PD1, a putative AdL/co-receptor, and their target cells that are exposed to IFN- γ began to upregulate PD-L1/B7-H1 [64, 65]. In fact, peripheral adaptation is to ensure tolerance of self-reactive immune responses that participate in tissue homeostasis [3] (Table 2). The adaptation model proposes that escape of T cells from medullary positive selection results in their apoptosis upon activation. This is evident during sepsis manifested as massive lymphopenia [66, 67] and immunodeficiency in aging populations, where there is a decline in naïve T cells [68], TcR repertoire diversity [69], and effective priming [70]. Sepsis, which induces thymic atrophy [71], has been observed to impede the development of autoimmunity [72], potentially due to the presence of defective T cells that escaped the medullary positive selection. Notably, these defective T cells express significantly lower levels of Bcl-xL, making them more sensitive to apoptosis [73]. Upon downregulation or loss of the AdRs, tissues become vulnerable to self-reactive immune responses or tumor cells expressing AdRs can tolerate anti-tumor immune responses. For instance, PD-L1 is mischaracterized as a ligand for PD-1 while it is a bi-directional receptor originally known as B7-H1, which upon engagement with PD-1, transmits survival signals via anti-apoptotic pathways, such as Bcl-xL induction in the target cells or tumor [74–78] (Fig. 1A). In fact, B7-H1 is a putative AdR, which upon blockade by immune checkpoint inhibitors, renders the tumor susceptible to apoptosis by T cells. Contrary to the view of PD-1 expressing T cells as purely exhausted, they are actively involved in tissue protection, producing TGF-β and relaying survival signals to PD-L1-positive target cells [79]. In fact, IFN-γ-producing T cells assist in tissue adaptation by upregulating B7-H1/PD-L1 on target cells, facilitating a protective immune environment [80]. Importantly, PD-1 signaling does not induce global immune inhibition, as p38 MAPK remains active, allowing specific regulatory functions to proceed [81]. Additionally, blockade of the PD-1/PD-L1 interaction inhibits - but does not completely abolish - IFN-γ production by T cells [82]. In fact, the efficacy of ICI is more related to the inhibition of survival signal in PD-L1 positive tumor cells rather than complete suppression of T cells. To this end, a higher efficacy of anti-PD1 compared to anti-PD-L1 could be because PD-1 can also bind CD28, which is a putative AdR being linked to Bcl-xL [61, 62]. Therefore, while anti-PD-L1 only blocks PD-1/PD-L1 pathway leaving PD-1/CD28 active (Fig. 1B), anti-PD-1 can block survival signal through both PD-L1 and CD28 (Fig. 1C). Importantly, melanoma and lung cancer express CD28 [83, 84], and its expression determines response to ICI in advanced NSCLC [85]. PD1 may have additional co-receptors on tumor cells that have yet to be discovered.

**Table 1:** Two-tiered positive selection of T cells during central immune adaptation

	Goal	Outcome	Defect	Function
**Cortex**	Selection of self-reactive T cells through MHC restriction and maturation into single-positive CD4⁺ or CD8⁺ T Cells	Maturation of T cells	Not reported	Support tissue homeostasis and integrity
**Medulla**	Selection of self-reactive T cells through survival of stress-inducing co-stimulatory signal II	Selection of functional T cells and elimination of defective clones	Lymphopenia

**Table 2:** Peripheral adaptation during homeostatic immune responses

	AdR	AdL/co-receptor
**Constitutive expression**	B7-H1/PD-L1 expression in the lungs, liver, kidneys, heart, pancreas, thymus, anterior chamber of the eye, and placenta	Expression of PD-1 on T_RM_ cells
**Induced expression**	IFN-γ-induced expression of B7-H1 on tumor cells	Expression of PD-1 on activated T cells

## Transient and chronic inflammatory immune responses aim to promote tissue integrity and adaptation: spontaneous tumor regression, immunosurveillance and immunoediting

The concept of transient and chronic inflammatory immune responses as mechanisms for promoting tissue integrity and adaptation is gaining clinical relevance. In wound healing, for instance, an initial inflammatory phase helps clear pathogens and damaged cells, setting the stage for a transition to homeostatic immune responses that support tissue repair and regeneration. Rather than viewing these responses simply as “pro-“ and “anti-inflammatory”, it may be more accurate to see them as phases in a dynamic process aimed at maintaining tissue health and adaptation to environmental changes. This dynamic is also evident in cancer biology, where early inflammatory responses involving M1 macrophages, Th1 cells, and CD8^+^ T cells target tumor cells and can induce significant tissue changes. Over time, however, there is a natural shift toward M2 macrophages and Th2 cells, which contribute to tissue remodeling, repair, and regeneration within the TME. Chronic M1/Th1 responses, while attempting to attack the tumor, can also create an environment that drives tumor evolution through the production of reactive oxygen species (ROS) and reactive nitrogen species (RNS), which cause DNA damage and genomic instability, promoting mutagenesis within the TME [86]. This process may alter the TME in ways that help host tissues adapt to and survive a carcinogenic environment. In some cases, this adaptation could lead to spontaneous tumor regression due to accumulated mutations that make tumor cells more prone to apoptosis or loss of proliferative potential. Clinical reports provide intriguing examples of this adaptive process leading to spontaneous regression. Melanoma, for instance, has well-documented cases of spontaneous regression, often associated with an immune response against melanoma cells [87]. Neuroblastoma, particularly in infants with stage 4S disease, has also shown spontaneous regression, potentially due to innate immune recognition or genetic factors that make the cancer less aggressive [88]. Renal cell carcinoma (RCC) has exhibited spontaneous regression, especially following infections or fever, which may trigger an immune response against tumor cells [89]. In lymphoma, infections, vaccinations, or immune-modulating events have been linked to regression [90]. Similarly, HCC has shown cases of regression, potentially due to immune-mediated responses, hypoxia, or vascular occlusion [91]. This adaptive immune role in disease progression also resonates with genetic examples where mutations offer survival advantages. The CCR5 Δ32 mutation provides resistance against HIV [92], while the sickle cell trait (HbS) mutation confers protection against *Plasmodium falciparum*, the parasite responsible for malaria [93].

Conversely, if the mutations are not beneficial to the host, they may drive tumor immunoediting and immune evasion, resulting in cancer progression (Table 3). Therefore, malignancy may be seen as a byproduct of the immune system’s adaptive efforts to remodel tissue in response to environmental toxicity [94]. On the other hand, M2/Th2 responses work to balance the mutagenic effects of inflammation by promoting repair and reducing tissue stress, thereby supporting tissue integrity and possibly limiting malignant progression.

**Table 3:** Transient and chronic inflammatory immune responses

	Function	Outcome
**Transient**	Elimination of nascent transformed cells	Tumor immunosurveillance
**Chronic**	Peripheral adaptation through the induction of mutations	* Spontaneous tumor regression * Tumor immunoediting

## Transforming cancer immunotherapy: moving beyond destruction to tissue-remodeling approaches

Clinical trials in cancer immunotherapy have revealed that while destructive approaches - such as CAR T cells, ICIs, bispecific T cell engagers (BiTEs), passive antibody therapies, allogeneic hematopoietic stem cell transplantation, and cancer vaccines - can initially shrink tumors, however they often lead to relapse as the TME adapts to evade immune detection (Table 4). For instance, therapies targeting the PD-1/PD-L1 pathway can induce significant initial tumor shrinkage but frequently fail to maintain long-term control as tumors develop immune evasion mechanisms [95]. Similarly, CAR T-cell therapy has demonstrated initial effectiveness in hematologic cancers, yet yields inconsistent results in solid tumors, where the immunosuppressive TME actively resists immune attacks, creating barriers to sustained tumor control and risking off-target effects in normal tissues [96]. Given these challenges, a shift toward tissue-remodeling immunotherapies is gaining traction. Instead of solely aiming for tumor destruction, this approach seeks to reprogram the TME and restore tissue integrity, leveraging the immune system’s inherent roles in tissue repair, regeneration, and immune surveillance. In such modulated TME, tumors are expected to shrink. Evidence of natural tumor dormancy in healthy individuals, as highlighted in recent reviews [97], suggests that maintaining tissue integrity may prevent cancer development from latent malignant cells. Chronic tissue injury, on the other hand, disrupts this balance, creating conditions conducive to cancer progression [98] - a phenomenon often likened to a “non-healing wound” [99]. For example, aging, UV radiation, and chronic irritants can impair skin homeostasis, promoting malignancy. Thus, restoring tissue integrity through immunotherapy could remodel the TME, making it hostile to tumor growth.

**Table 4:** Cytotoxic and homeostatic immunotherapies

	Immunotherapies	Function	Outcome
**Cytotoxic**	CAR T cells, ICI, BiTE, CT, cancer vaccine	Maturation of T cells	* Prolong survival * Off-target toxicity, GVHD * Tumor recurrence and immune evasion * Secondary cancer
**Homeostatic**	* Correcting tissue microbiome dysbiosis * Extracellular vesicle transplantation * Harnessing homeostatic T_RM_ cells * Targeting predominant tumor-specific AdRs	Remodel TME	Tumor elimination or permanent tumor dormancy

Developing tissue-remodeling immunotherapies will require a comprehensive understanding of the tissue-resident immune system and its complex network of interactions. Current clinical data on cancer immunotherapies, however, are largely based on reductionist, cytotoxic-focused approaches. Advances in big data and spatial transcriptomics now enable a systems immunology perspective, capturing tissue immune dynamics at a single-cell resolution. This shift could enable the creation of therapies that leverage tissue-resident immune functions to restore tissue homeostasis, moving closer to curative treatments. Recent findings in hepatic immune networks, for instance, suggest that the collective function of the hepatic immune system is orchestrated through dominant-subdominant interaction network; therefore, targeting dominant functional immune cells within a complex, could drive therapeutic tissue remodeling and contribute to sustained cancer control [100].

Exploring new pathways for homeostatic immunotherapy holds significant promise for advancing cancer treatment (Table 4). One promising approach to restoring tissue integrity is the correction of microbiome dysbiosis, a key regulator of immune function. Fecal microbiota transplantation (FMT) has shown potential in improving the efficacy of ICIs [101, 102], though challenges such as infection risks from donor microbes and limited efficacy of pre-transplant antibiotics [103], donor selection, microbiome variability and durability, as well as regulatory and safety concerns limit the feasibility. These limitations highlight the need for innovative immunotherapeutic strategies that not only remodel the TME, but also correct microbial imbalances. For instance, Th9 cells, known to produce the tissue-repairing factor amphiregulin, have been shown to reduce allogeneic transplant toxicity by preserving graft-versus-leukemia effects while mitigating graft-versus-host disease [17]. Skin-resident CD8^+^ T cells also contribute to tissue remodeling by expressing amphiregulin and critical cytokines such as IFN-γ and TNF-α [45]. Similarly, IL-17-producing γδ T cells in the skin participate in tissue integrity by producing amphiregulin, which is essential for barrier function [104]. Additional strategies to correct microbiome dysbiosis and enhance tissue integrity include soluble IgA, which helps balance gut microbes and protect the epithelial barrier [105], and extracellular vesicles (ECV) derived from healthy tissue cells [106, 107], which may hold the potential to mediate tissue remodeling immune responses. Future breakthroughs in homeostatic immunotherapy could arise from better understanding the molecular pathways by which T_RM_ cells support tissue repair, the immune mechanisms driving spontaneous tumor regression, and strategies for targeting dominant tumor-associated AdRs that sustain cancer resilience. Each tissue or organ is likely to express tissue-specific dominant AdRs that have yet to be discovered.

## Data Availability

No new data were created or analyzed in this study. Data sharing is not applicable to this article
